# Governance of community health worker programs in a decentralized health system: a qualitative study in the Philippines

**DOI:** 10.1186/s12913-021-06452-x

**Published:** 2021-05-12

**Authors:** Warren Dodd, Amy Kipp, Bethany Nicholson, Lincoln Leehang Lau, Matthew Little, John Walley, Xiaolin Wei

**Affiliations:** 1 School of Public Health and Health Systems, University of Waterloo, 200 University Ave. W, Waterloo, Ontario N2L 3G1 Canada; 2 International Care Ministries, Manila, Philippines; 3 School of Public Health and Social Policy, University of Victoria, Victoria, Canada; 4 Nuffield Centre for International Health and Development, University of Leeds, Leeds, UK; 5 Dalla Lana School of Public Health, University of Toronto, Toronto, Canada

**Keywords:** Community health workers, Universal health coverage, Health systems, Local government, Gender, Philippines

## Abstract

**Background:**

Community health worker (CHW) programs are an important resource in the implementation of universal health coverage (UHC) in many low- and middle-income countries (LMICs). However, in countries with decentralized health systems like the Philippines, the quality and effectiveness of CHW programs may differ across settings due to variations in resource allocation and local politics. In the context of health system decentralization and the push toward UHC in the Philippines, the objective of this study was to explore how the experiences of CHWs across different settings were shaped by the governance and administration of CHW programs.

**Methods:**

We conducted 85 semi-structured interviews with CHWs (*n* = 74) and CHW administrators (*n* = 11) in six cities across two provinces (Negros Occidental and Negros Oriental) in the Philippines. Thematic analysis was used to analyze the qualitative data with specific attention to how the experiences of participants differed within and across geographic settings.

**Results:**

Health system decentralization contributed to a number of variations across settings including differences in the quality of human resources and the amount of financial resources allocated to CHW programs. In addition, the quality and provider of CHW training differed across settings, with implications for the capacity of CHWs to address specific health needs in their community. Local politics influenced the governance of CHW programs, with CHWs often feeling pressure to align themselves politically with local leaders in order to maintain their employment.

**Conclusions:**

The functioning of CHW programs can be challenged by health system decentralization through the uneven operationalization of national health priorities at the local level. Building capacity within local governments to adequately resource CHWs and CHW programs will enhance the potential of these programs to act as a bridge between the local health needs of communities and the public health system.

**Supplementary Information:**

The online version contains supplementary material available at 10.1186/s12913-021-06452-x.

## Background

Sustainable Development Goal (SDG) 3 commits to promoting wellbeing “for all, at all ages” by the year 2030, with SDG 3.8 specifically committing to universal health coverage (UHC) [1]. This emphasis on universal health coverage has strengthened national and international commitments to the equitable delivery of primary health care services, especially in low-resource settings, and has renewed interest in community health worker programs as a means of implementing UHC [2]. Community health worker (CHW) programs, in which community members are trained in basic health care delivery to provide care to their local communities, have been identified as a key intervention for achieving UHC [2–5]. Depending on the program, CHWs may be responsible for a wide range of tasks (e.g., maternal and childhood health, nutrition, immunizations) or for individual disease-specific interventions (e.g., anti-malaria campaigns, tuberculosis testing) [6]. In low- and middle-income countries (LMICs) where governments are increasingly adopting decentralized health care policies, CHWs are often the first point of contact with health systems for many individuals [6, 7]. Thus, these programs serve as an intermediary between health systems and communities, with the intention of extending the reach of health care providers, enhancing access and equity of health services, and improving individual and community level health outcomes (e.g., [4, 6, 8–12]).

Despite this optimism for CHW programs and their potential contributions to achieving UHC, there are several factors that may negatively influence the governance of these programs.^1^ In many LMICs, local, regional, and national politics and policies have been found to impact the effectiveness of CHW programs [9, 13]. For example, the existence of legislation regarding CHWs can support the rights of CHWs (e.g., payment, working conditions) and enhance the sustainability of programs [9]. Conversely, political and local support can be weak, with limited human and fiscal resources allocated for programs, minimal training and supervision provided to CHWs, and inconsistent monitoring of programmatic outcomes [11, 14–16]. Additionally, operating at the intersection of formal health systems and communities necessitates the involvement of multiple actors, but relationships between these actors are not always coordinated or strategic [12, 17, 18]. These relationships, which are influenced by factors such as a community’s socioeconomic status, the level of community participation, and the power of community leaders, can complicate program governance [6, 12, 15].

Sociocultural and individual-level factors also influence the governance of CHW programs. Individuals have diverse backgrounds and motivations for becoming and remaining CHWs, which are based on socio-economic, political, and pro-social incentives (e.g., civic and religious ideals of service and care) [19–21]. For example, existing research has explored the role of gender and household socio-economic status within CHW programs [9, 11, 21, 22]. These factors can also influence CHW training, retention, and remuneration [6, 14, 16]. Although existing literature has explored these sociocultural and individual-level factors, as well as the broader governance challenges of CHW programs, few studies have examined the intersection of these factors and their potential influence on the contributions of CHW programs to achieving UHC within LMICs.

### The community health worker program in the Philippines

The Philippines, along with the 192 members states of the United Nations, committed to achieving the 17 SDGs in September 2015 [1]. In 2019, building on decades of health care sector reform, President Rodrigo Duterte signed the Universal Health Care Act bringing the Philippines one step closer to realizing SDG target 3.8 of achieving UHC. The goal of the Universal Health Care Act was to provide Filipino citizens with equitable access to the full continuum of health services [23]. As CHW programs have globally been identified as an important component of UHC [24], this new policy in the Philippines provides an opportunity to explore the country’s CHW programs and their potential contributions to operationalizing UHC.

Two main CHW initiatives exist in the Philippines: *Barangay* Health Workers (BHWs) and *Barangay* Nutrition Scholars (BNSs).^2^ Both groups consist of volunteers or low-paid workers who deliver or refer patients to primary health care services in communities. BHWs provide information, support, and referral services on topics such as maternal and child health, family planning, immunizations, and disease-specific care (e.g., malaria, tuberculosis) [26]. The BNS program focuses primarily on implementing nutrition related initiatives with malnourished infants and children (0–5 years), as well as to nutritionally vulnerable pre- and postnatal women [27]. For the purposes of this study, we collectively refer to BHWs and BNSs as CHWs because of the shared characteristics across the two groups.

**Fig. 1:**
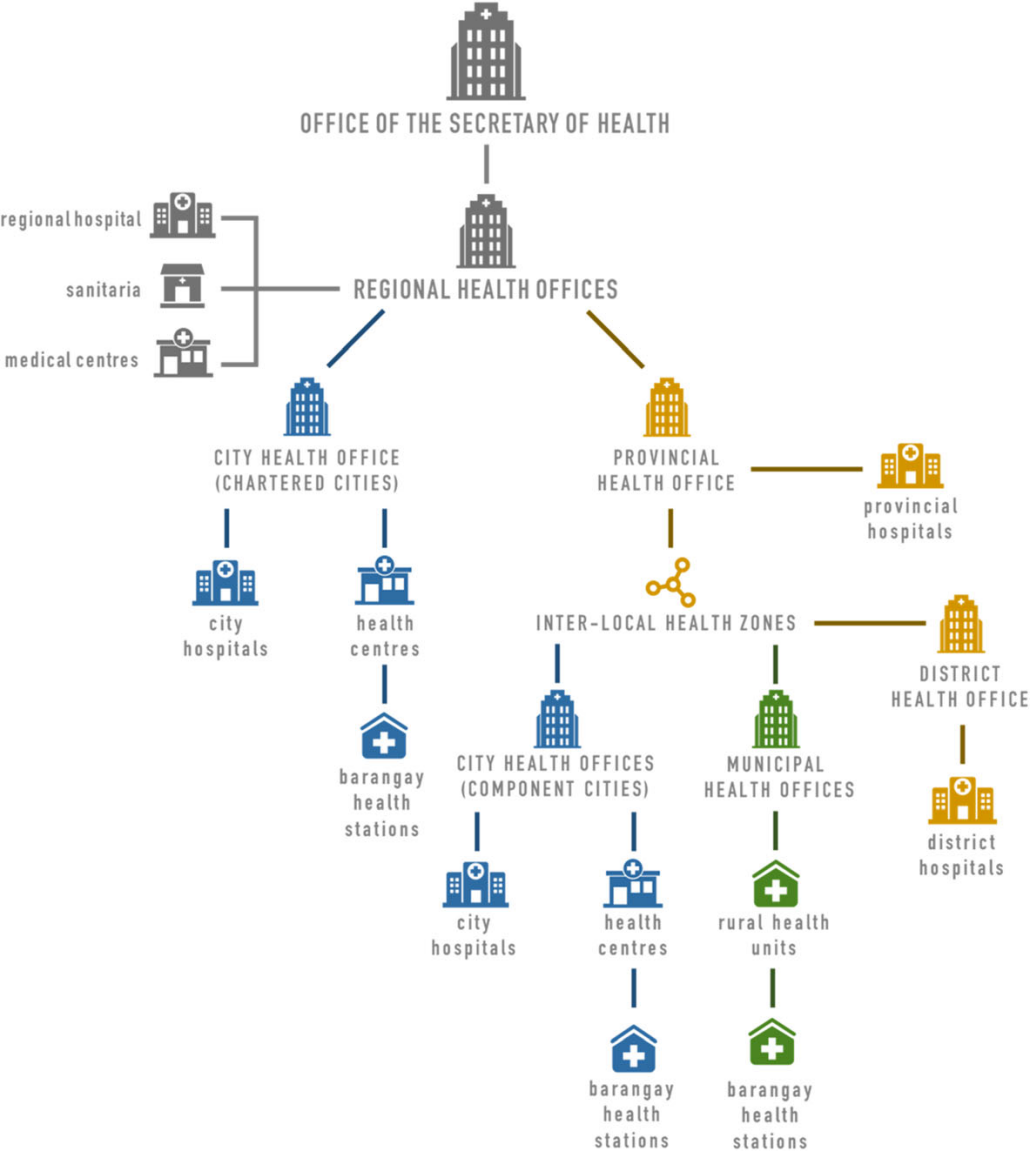
Governance structure of the Philippines’ decentralized health system (adapted from [33, 34])

In the Philippines, CHW programs were promoted as one component of the country’s move toward health care decentralization. In the 1990s, the Government of the Philippines moved to a decentralized governance system, enacting the Republic Act No. 7160, known as the 1991 Local Government Code [28–30]. One main outcome of the Local Government Code was the devolution of the governance and administration of health care services from the national government to local government units (LGUs) [29–32]. With this shift, the national government, through the Department of Health (DOH), maintained responsibility for high-level health governance decisions, such as setting and enforcing the national health policy agenda [28, 29]. Provincial governments were tasked with tertiary level health services, including the operation of provincial and some district hospitals [28, 29]. Smaller municipalities were made responsible for the delivery of primary and preventive care through rural health units and *barangay* health stations. Governments of large, urbanized cities were given responsibility for managing city hospitals, health centres, and *barangay* health stations within their city boundaries [28, 29] (see Fig. 1).

The DOH sets the national agenda for CHW programs. The government of the Philippines outlines that CHWs should be trained through an accredited government or non-governmental organization, as well as incentivized through an allowance, training opportunities, and other benefits [35]. According to the Republic Act no. 7883 (1995), often referred to as the *Barangay* Health Worker Act, BHWs are to receive 6 months of training and undergo a post-training accreditation process implemented by the DOH, local governments, and the Civil Service Commission. BNSs are to receive in-class training followed by a 20-day practicum under the supervision of the District or City Nutrition Program Coordinator [27]. LGUs ultimately govern CHW programs that operate through city health centres, rural health units, and *barangay* health stations [36]. In some instances, CHWs are employed by a specific *barangay*, while other CHWs are employed by municipal or regional governments. A CHW may also be simultaneously employed by multiple levels of government (e.g., both a *barangay* and a city).

CHWs have been described as the front-line of health care delivery in the Philippines [37]. Existing studies have highlighted the potential benefits of Filipino CHWs in delivering postpartum health care [36], supporting malaria control [38], administering diagnostic tests for HIV and tuberculosis (TB) [39], and implementing national nutrition programs [40]. Additionally, several studies have identified CHW programs as critical to addressing health service delivery challenges in the Philippines, particularly in rural areas [26, 36, 38].

However, the effectiveness of CHW programs in improving health outcomes in the Philippines has been questioned, with some research highlighting how limited training, resources, incentives, and community support hinders the success of CHW programs [38, 39]. Research has also highlighted the gender imbalance of CHWs in the Philippines, where the majority of volunteers identify as women [22]. As demonstrated in other contexts (e.g., [21]), the reliance on gendered notions of care in CHW programs may contribute to a structural lack of recognition and an undervaluing of CHWs.

These challenges must also be understood in the context of the country’s decentralized health system. In the Philippines, decentralization is considered by some to contribute to the democratization of health care by enhancing community participation, responsiveness to local problems, and the inclusion of marginalized perspectives [28, 31]. However, achieving these potential benefits is a complex task, with some health care workers and non-governmental organizations (NGOs) regarding the existing devolution of health care in the Philippines as “ineffective in improving access, efficiency and quality of health services” ([41], p.61). Decentralization in the Philippines is also critiqued more broadly for minimizing the role of the state, enhancing inequality across communities, and strengthening the power of potentially corrupt local political leaders [31]. Although existing work has highlighted decentralization as the backdrop of CHW programs, little research has explored the ways in which a decentralized health system directly impacts the governance of CHW programs and the experiences of CHWs within decentralized health systems.

In this context of health system decentralization and the push toward UHC in the Philippines, the objective of this study was to explore the experiences of CHWs and how the governance and administration of CHW programs shaped these experiences across different settings. We brought together the perspectives of CHWs and administrators of CHW programs to examine individual-level and structural factors that influenced the operations of CHW programs in two provinces. Overall, we assessed how these factors interacted and shaped the well-being of CHWs, the role of non-state actors in CHW programs, and the governance and administration of CHW programs.

## Methods

### Study location

**Fig. 2:**
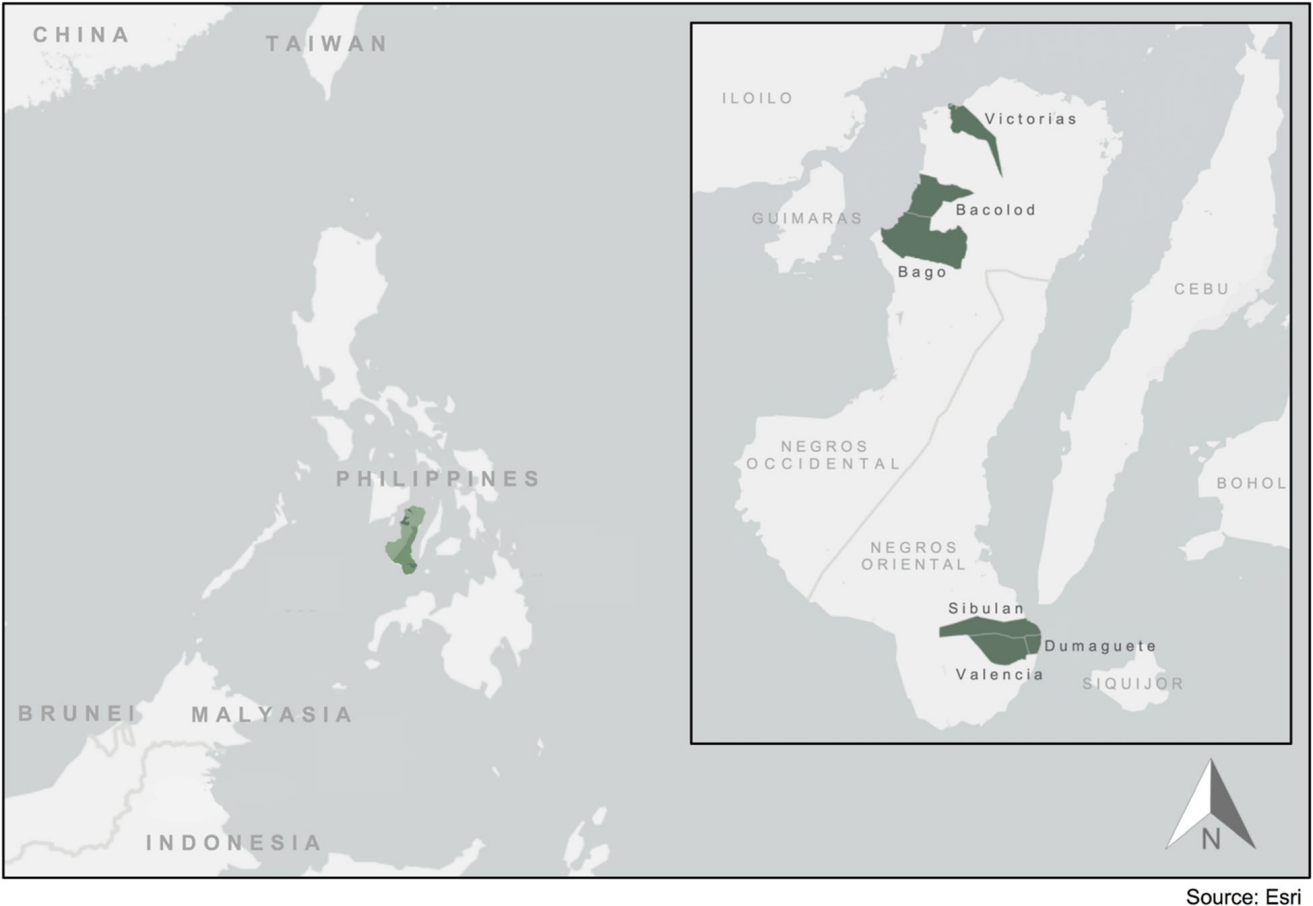
Map of the study region: Negros Occidental (Bacolod, Bago, and Victorias) and Negros Oriental (Dumaguete, Valencia, and Sibulan), Philippines

This study was conducted in the provinces of Negros Occidental and Negros Oriental. In Negros Occidental, we conducted interviews in 15 *barangays* across three cities (Bacolod, Bago, and Victorias). In Negros Oriental, we conducted interviews in 21 *barangays* across three cities (Dumaguete, Valencia, and Sibulan) (see Fig. 2). The *barangays* included in the study were primarily located in urban or peri-urban settings. Given the decentralization of the health system, the study was conducted across multiple *barangays*, cities, and provinces to facilitate comparisons across settings.

### Data collection

We developed a semi-structured interview guide to explore various factors related to the experiences of CHWs within the context of a decentralized health system (see Table S1 in the supplementary file for semi-structured interview guide). As described above, CHWs (*n* = 74) included both BHWs and BNSs. Interview questions with CHWs focused on both individual-level motivations, training, and competencies of CHWs, in addition to structural and political factors that shaped CHWs’ experiences of their work. To provide a broader context of how CHW programs operated across different settings, we also interviewed CHW administrators (*n* = 11), which included individuals in a supervisory capacity over CHWs (midwives (*n* = 3), nurses (*n* = 2), community health officers (*n* = 2), and a *barangay* captain (*n* = 1)).^3^ Given the different roles held by these individuals, interviews with CHW administrators were open-ended and focused on the organization, supervision, and governance of CHW programs.

Recruitment occurred in person at *barangay* health stations, rural health units, city health centres, local government offices, and CHW administration offices. Recruitment was initially facilitated through pre-existing relationships between health facilities and our local community partner organization. We then used a snowball sampling approach whereby we asked interviewees if they could connect us with other individuals who might be willing to participate in the study.

Interviews were co-facilitated by a member of the research team and a research assistant. The interview guide was piloted in two *barangays* with CHWs and CHW administrators. Following this piloting phase, the guide was clarified and refined. Interviews were conducted in the preferred language of the interviewee (English, Hiligaynon, Cebuano) and were audio recorded. For interviews not conducted in English, the research assistant provided real time translation to assist with interview facilitation. Prior to conducting an interview, a description of the study was provided, and written or verbal informed consent was obtained from all participants.

Audio recordings were transcribed to facilitate data analysis. For interviews that required translation, non-English excerpts were translated to English during the transcription process.

### Data analysis

Thematic analysis was used to analyze the data collected from the semi-structured interviews (e.g., [11, 21, 39]). An open coding approach was used to inductively identify initial categories within the data using the data analysis software NVivo. The data were categorized based on frequently discussed components of the CHW programs – such as training, remuneration, recruitment, and individual motivations – as well as the role of various actors within these programs (e.g., CHWs, program administrators, *barangay* captains, NGOs, etc.). We also paid specific attention to the differences and similarities of participants’ experiences with the CHW programs within and across geographic settings. We collaboratively developed these initial categories into broader themes and then returned to the data to inductively code for emergent themes (e.g., variation in program administration, local politics, the role of care). Additionally, basic demographic (e.g., age, education, length of time as a CHW) and training (e.g., length of basic training, training provider by region) information was extracted from interview transcripts and descriptive statistics were calculated.

## Results

### Demographic factors of CHWs

The CHWs in our study almost all identified as women (one male participant), with the majority (*n* = 55; 74.3%) between the ages of 40–69 years old, and an average age of 48.5 years (range: 27 years–76 years) (see Table 1). Through the interviews, several CHWs highlighted age as a factor influencing their experiences. For example, a CHW from Bacolod City in her sixties explained that “young people don’t want to be trained without allowance,” whereas many older CHWs told of volunteering for years before receiving any remuneration for their work. Additionally, CHWs identified factors such as having a young family or being enrolled in school as limiting the ability of the younger generation to work as CHWs.

**Table 1:** Age and length of employment among community health workers in Negros Occidental and Negros Oriental, Philippines (*n* = 74)

**Age group**	**n (%)**
20–29 years	4 (5.4%)
30–39 years	12 (16.2%)
40–49 years	20 (27%)
50–59 years	22 (29.7%)
60–69 years	13 (17.6%)
70–79 years	3 (4%)
**Time employed**	**n (average age)**
< 1 year	12 (37.5 years)
1–5 years	13 (41.9 years)
6–10 years	10 (47 years)
11–15 years	9 (50.7 years)
16–20 years	9 (53 years)
21–25 years	11 (61 years)
26–30 years	3 (65.7 years)
31–35 years	7 (60 years)

To better understand the educational backgrounds of CHWs, we asked participants about their highest level of education. Overall, 47.3% (*n* = 35) of participants had college or vocational college education, followed by 39.2% (*n* = 29) of participants who had high school education. Of those who attended college or vocational college (*n* = 35), the most common fields of study were business and commerce (*n* = 7; 20%), secretarial studies (*n* = 6; 17.1%), and education (*n* = 4; 11.4%). A minority of participants had training in either health care (*n* = 3; 8.6%) or social work (*n* = 3; 8.6%) (see Table 2).

**Table 2:** Level and field of education among community health workers in Negros Occidental and Negros Oriental, Philippines (*n* = 74)

**Highest level of education (*n*** **= 74)**	**n (%)**
College	32 (43.2%)
Vocational college	3 (4.1%)
High school	29 (39.2%)
Elementary school	8 (10.9%)
No response	2 (2.7%)
**Field of study in college or vocational college (*n*** **= 35)**	**n (%)**
Business or commerce	7 (20%)
Secretarial studies	6 (17.1%)
Education	4 (11.4%)
Midwifery or nursing	3 (8.6%)
Computer sciences	3 (8.6%)
Social work or psychology	3 (8.6%)
Hospitality	2 (5.7%)
Other	6 (17.1%)
No response	1 (2.9%)

### The role of care and social relationships in motivating and shaping CHW experiences

Many CHWs explained that they were motivated to become and remain CHWs because of their compassion, love, and desire to serve their communities. A woman from Bacolod City illustrated these characteristics explaining that in her role as a CHW, she often purchased medicine and rice for community members when she found they had insufficient resources. She explained that she provided these resources because of her love for the community. Similarly, another participant from the same city who had been working as a CHW for nearly 30 years, explained how her love of the role motivated her to continue as a CHW, stating, “if there are poor patients, then we are eager to help them […] if I do not love [this work] then I [would] have left.”

When asked about the background or credentials needed to be a CHW, many participants explained that there were limited requirements, and instead focused on ideal characteristics of CHWs. These characteristics included being compassionate, patient, respectful, dedicated, and approachable. A CHW from Victorias City explained that “as long as she want[ed] to help other people” she could serve as a CHW. Another CHW from the same city, with a background in education explained that although being a CHW was “far from the program” that she received her training in, she was successful in the role because her “interest [was] to serve the people.” Moreover, several CHWs identified their relationships with community members as central to the role. For example, in Bago City a participant in her seventies who had served as a CHW for over 30 years explained that she wished to retire, but was persuaded to continue working because, “the people here say that if new BHWs will come, they cannot trust them.” Others explained that if you were a BHW you were “only thinking of others” and must act as a “role model” for the community.

Social relationships also played a key role in the process of recruiting and retaining CHWs. Most CHWs were either appointed directly by a political leader (i.e., the *barangay* captain or mayor), or recruited by individuals working in health care (i.e., doctor, midwife, nurse, or another CHW). In many cases, CHWs were also related to or friends with those who appointed or recruited them. One participant from Victorias City highlighted the connection between social relationships and care, and explained that “she was recommended by the people in the community” because she was liked for being “so accommodating,” “friendly,” and “compassionate” and because she was “always assisting and helping in people’s needs.” Thus, building and maintaining positive and often caring relationships with community members was seen as integral to the CHW role.

When discussing their low remuneration and, in some cases, willingness to volunteer, participants also emphasized the central role of care. One CHW from Bacolod City explained that although her stipend may be taken away with the change of *barangay* administration, she was willing to continue to volunteer as a CHW so long as she was able to “help people in the community […]. She explained, “it’s not about the salary, it’s about compassion and care.” Many others echoed a similar sentiment of care. For example, a CHW in Sibulan recounted the enjoyment she experienced in her work, explaining, “we are happy to serve […] even though we receive a small amount of allowance, still we are happy.” Similarly, a CHW in Victorias City described her willingness to serve people in need, stating, “when you truly serve the public, even though you are not being paid, as long as people need you then, you are willing to serve.”

### Governance challenges of administering CHW programs in a decentralized health system

Based on the experiences of CHWs and program administrators, we found that the decentralization of the health system contributed to a number of administrative challenges in the governance of the CHW program. A common theme throughout the interviews was that the administration of the CHW program varied across and within regions. More specifically, participants discussed how their experiences of human and fiscal resources, in addition to their training, was influenced by the *barangay* or region where they worked.

#### Human and fiscal resources

When discussing the remuneration of CHWs, a program administrator from Bago City explained that “it just depend[ed] [on] the income of the *barangay*.” Similarly, a CHW in Valencia believed that having a prominent source of tax revenue within a *barangay*, such as a large corporation**,** enabled officials to *“*afford to give more to the BHWs.” In one *barangay* in the region, where an energy corporation based its operations, this participant explained that CHWs were hired through “job orders” and received a regular salary. However, in *barangays* without this source of tax revenue, CHWs were appointed on a more voluntary basis and received only a small stipend for their work.

In terms of CHWs’ workload, participants’ experiences also varied greatly by region. One CHW in Bago City explained that the existing number of CHWs were “not enough,” stating that in her *barangay*, each CHW was responsible for providing care to five *puroks,* while in other *barangays* she knew of one CHW was responsible for one *purok*.^4^ She saw this workload was directly related to her *barangay’s* limited budget, explaining, “they would like to add more BHWs for the loads to become easier, lighter, but […] there’s no allowance for the new ones, that’s the problem.” However, in Dumaguete a CHW told of her very different experience, stating that she often had the ability to help out at a neighbouring *barangay* due to a small workload. She explained, “if I stay in [my] *barangay*, I have nothing to do there because most of the people living there are already well-off […] that’s why we have very little number of patients.”

Several CHWs highlighted consequences associated with limited fiscal resources. For example, one CHW in Victorias City explained that she frequently paid for program expenses (e.g., printing costs) out of her “pocket.” She stated, “it depends on the national budget for the *barangay.* Sometimes it doesn’t fit everything.” Additionally, a program administrator in Dumaguete explained that the structure of the budgets in some *barangays* limited the allocation of resources for specific health concerns (e.g., nutrition), which disrupted the delivery of health programs by CHWs.

Although the availability and administration of resources in individual *barangays* greatly influenced how the CHW program was experienced, non-state actors (i.e., non-governmental organizations (NGOs) and individual CHWs) also influenced the implementation and effectiveness of the CHW program. For example, to help address a lack of resources, an administrator in Bacolod City explained that she would often encourage CHWs to partner with NGOs to expand the reach of their child nutrition programs. She explained that she viewed such partnerships as a “golden opportunity to actually establish a healthier *barangay*, a healthier community.” Additionally, individual CHWs played a role in advocating for greater health resources at the *barangay* level. This advocacy was illustrated by a CHW in Sibulan who stated: “I asked for a budget but they said that there is no budget […] so I will [continue to] ask, ‘can we put a budget for the health, especially for the feeding of the malnourished children?’ I will try, I will try, I will try.”

#### Training length and providers

CHWs and administrators identified several common characteristics of CHW training across cities including some level of basic training, yearly ‘refresher’ trainings, and monthly ‘update’ meetings. However, components of CHW training, including the length, fees paid to attend training, providers of basic training, and the availability of supplementary training, varied between regions and, in some cases, within *barangays.* In Negros Occidental, CHWs and administrators (*n* = 43) stated that basic training ranged from 1 day to 3 weeks, with 1 week of training mentioned most frequently (*n* = 19; 44.2%). In contrast, there was greater variability in the length of basic training reported among CHWs and administrators from Negros Oriental (*n* = 42), with these participants sharing that basic training lasted anywhere from 1 day to 3 months.

The training provider also varied between regions, with some participants mentioning multiple organizations involved in CHW training. Of the 43 CHWs and administrators from Negros Occidental, 79.1% (*n* = 34) of these participants stated that city health centres provided some form of training. However, of the 42 CHWs and administrators from Negros Oriental, only 31.0% (*n* = 13) mentioned city health centres as a training provider. Conversely, the local Disaster Risk Reduction Management Office was a frequently mentioned training provider in Negros Oriental (*n* = 16; 38.1%), but rarely mentioned as a training provider in Negros Occidental (*n* = 2; 4.7%). Non-state actors, such as NGOs, also played a role in training. In Negros Occidental, 34.9% (*n* = 15) of participants stated that NGOs were a training provider, whereas 16.7% (*n* = 7) of participants in Negros Oriental indicated that NGOs provided training.

Several CHWs explained how NGO training supplemented their CHW basic training. In one case, when asked if she had been trained on specific diseases, such as tuberculosis and diabetes, a CHW in Victorias City responded, “during my training, I was taught just a little bit about those disease [s] but fortunately I was trained by [an] NGO […] about those diseases and how to assist.” She explained that she was “very thankful” for the NGO training because she now had the knowledge to manage different diseases. In another case, two CHWs from the same *barangay* in Bacolod explained that an NGO provided financial sponsorship for their training, which had been conducted by the nearby city health office.

Individual agency was also an important factor in shaping how CHWs accessed additional training, with some CHWs describing their efforts to seek out training opportunities to better serve their communities. In other cases, more experienced CHWs explained that they frequently acted as informal mentors to new CHWs who had not yet received their basic training.

### Governance challenges and local politics

As previously mentioned, CHWs could be employed by multiple levels of government (e.g., *barangay* and city). Depending on the context, when a CHW was simultaneously employed by multiple levels of government, they often engaged in different tasks in these different roles (e.g., community outreach and health promotion at the *barangay* level; health care administration at the city level). In addition, these CHWs often received compensation for both roles, with the salary from the city level frequently higher than the salary from the *barangay* level.

For CHWs who were employed at only the *barangay* or city level, local politics, including the power of local political leaders, played an important role within the administration of CHW programs. These CHWs explained that their positions were frequently dependent on whether they supported the current *barangay* captain or mayor. In many instances, if the *barangay* or city administration changed, existing CHWs who did not support the new administration were replaced. One CHW from Bacolod City provided an example of this situation, stating, “if our *barangay* captain did not win and the new *barangay* captain is in the position, there is a possibility that he will change the BHWs with the new ones whom he promised to be hired.” Highlighting the power of local leaders, another CHW working in Sibulan stated, “when the mayor doesn’t like you, then you will be out.” Another CHW from Bacolod City explained, “my *barangay* captain candidate won so I will be paid by the *barangay* captain and the city […] But if my *barangay* captain […] lost I would not be paid by the *barangay* […].” This cycle of replacing CHWs with the change of administration also had consequences for ensuring workers were adequately trained in their roles. Several administrators spoke of this challenge, explaining that it led to conducting more frequent trainings, despite having the same, limited budget.

In many cases, the power of local political leaders (i.e., the power of the *barangay* captain or mayor) enhanced the precariousness of the CHW position. This was exemplified by one program administrator in Valencia who reflected, “job security is relative to your political stance.” If CHWs were employed by both *barangays* and regional levels, their position as a CHW was more secure than CHWs who were only employed at the *barangay* level. If CHWs did not support the *barangay* captain or mayor they may be replaced at the local level, which might result in a reduction to their income.

CHWs and program administrators discussed navigating these local politics in a number of ways. Some CHWs mentioned the security that came from becoming an accredited CHW, a process they described as involving: completing basic training; working voluntarily as a CHW for 1 year; receiving a reference from a midwife or NGO representative; and receiving a reference from the head of their local CHW association. Participants explained that local political leaders could not replace them once they received accreditation. For example, two CHWs working in Bacolod City explained, “if the mayor changes and if you are not accredited, there’s a big chance that you will be replaced,” and “accredited BHWs can stay here forever, despite administration changes, but [non] accredited BHWs and BNSs [can] be pulled out if new administration arrives […].” However, another CHW from the same region also explained that in order to be accredited, CHWs had to receive an endorsement by their local political leader. Several CHWs discussed how being CHWs, therefore, impacted their personal politics. In a different interview in Bacolod City, one CHW stated, “[during elections] we always support the administration, always […].” In Bago City, another CHW described how she and other CHWs navigated this system by remaining non-partisan during elections. She explained, “you will have the chance to be pulled out from the position [if you campaign for the opposition] so that’s why we are non-partisan.” Additionally, several administrators spoke about lobbying for CHWs when a new administration wanted to replace them. One program administrator from Bacolod City explained that “they can just lobby especially for better performing BNSs […] or submit a letter of appeal.” However, she continued to explain that she had yet to be successful in changing the outcome when a new administration replaced existing CHWs.

## Discussion

### A reliance on care as a motivation to retain workers can create health system vulnerabilities

There are purported benefits of a workforce motivated by care and situated within communities such as enhanced local legitimacy, a sustained commitment to achieving positive health outcomes, and localized knowledge of community needs and health systems [8, 11]. Indeed, these benefits are central to why CHWs are lauded in their role as a bridge between local communities and public health systems across many contexts. There are also several benefits of decentralization for empowering community members to become participants in health care delivery. By participating in CHW programs, CHWs motivated by care may experience a sense of fulfilment, enhance their connections and networks, improve their status within their communities, and gain new health-related skills and knowledge [42, 43].

However, as demonstrated through our research, the care work that CHWs engaged in was also directly related to their identities (e.g., gender and age). Although we did not specifically ask participants how their identities shaped their experiences as CHWs, several participants suggested that younger individuals were less willing to work for low wages. This resulted in some older CHWs working past their expected retirement dates. Additionally, all but one of the CHWs interviewed identified as women which highlights an important gendered dimension to the Philippines’ CHW programs, as seen in other CHW programs in LMICs [42, 44–46].

It is important to consider the potential, and often unintended consequences of gender and other sociocultural factors in shaping the experiences of CHWs [42, 46]. For example, a study exploring the Philippines’ BHW program suggested that the feminized nature of the program limits BHWs’ duties to those perceived as an extension of existing ‘mothering’ and ‘caring’ roles [43]. Similarly, a study of a maternal health CHW program in Rwanda found that women CHWs often performed care work that went beyond their volunteer responsibilities (e.g., paying for patients’ transportation) which had the potential to undermine the sustainability of the program [44]. Additionally, both gender and caste have been found to impact the effectiveness of the Pakistan Lady Health Worker (LHW) program, as local norms discourage women from entering the homes of unrelated men or travelling beyond specific caste-based boundaries [46].

Such realities may create vulnerabilities for the recruitment and retention of CHWs, and health systems more broadly, for example if CHWs are either undervalued or underpaid (i.e., do not receive adequate remuneration) for the essential care they deliver. In societies where care work is feminized, it is important to acknowledge, and correct, existing gender pay gaps when allocating resources for CHW programs to ensure that the work and time of CHWs are appropriately valued [42]. As resources are leveraged in the push toward UHC in the Philippines and other contexts, resource allocation should reflect the important role of CHWs within the broader health system.

### A decentralized health system can create challenges for CHWs

The devolved responsibility of health care delivery from the national to the local level contributed to regional variations in the administration of CHW programs. In some cases, this localized control resulted in inadequate or inconsistent human and fiscal resources between *barangays* and municipalities. There was also variation in the training provided between *barangays* and municipalities, with CHW training influenced by whether and how local governments prioritized health-related expenditures. In some areas, rather than a standardized basic training provided by local governments, NGOs or more experienced CHWs provided training to new CHWs. Importantly, this lack of standardized training may influence the ability of CHWs to adequately or consistently understand and operationalize their roles and responsibilities. This lack of standardized training may contribute to community members having low confidence in CHWs, which in turn may affect if and how these individuals trust or access care from CHWs [39]. These findings support those of Mallari et al. (2020) who found that the decentralization of health care delivery had material consequences for the daily work experiences of BHWs, including their scope of work, incentive packages, training and supervision, and material supports, which can have consequences for the overall effectiveness of CHW programs [43]. As previously discussed, it is also important to view these findings in the context of how gender and other sociocultural factors shape CHW experiences including access to resources and training. More specifically, by positioning this work as an extension of existing ‘mothering’ or ‘caring’ roles [43], this may create situations where specific resources or training are overlooked or deemed unnecessary for CHWs to effectively engage in their work.

Moreover, our findings demonstrate the ways in which local politics challenge the governance of CHW programs. Local political leaders, such as *barangay* captains and mayors, play a key role in appointing and replacing CHWs, as well as implementing program budgets. This concentration of power locally can have several implications for the effectiveness of CHW programs (e.g., [47, 48]). As mentioned by several CHWs, there was a perceived need to align themselves with the current local administration in order to maintain their jobs. Indeed, many of the CHWs in our study had served in their roles for over 10 years, perhaps indicating that some of these individuals had shifted their political affiliation over time to support changing political leaders. Alternatively, and as discussed later, the accreditation of some CHWs provided a buffer against the influence of local politics and contributed to the longevity of these CHWs.

In addition, the frequent turnover of CHWs, which was often prompted by a change in local administration, made it difficult to ensure that CHWs were adequately trained. This turnover also resulted in more frequent trainings, which had the potential to drain local health budgets as well as human resource capacity. Moreover, the social relationships and trust between CHWs and community members, identified as central to the functioning of the CHW model [5, 6, 9, 11] may be eroded with the frequent replacement of CHWs. For example, Mallari et al. (2020) found that the turn-over of BHW staff can negatively affect community members’ perceptions of the program, the embeddedness of BHWs in the community, as well as the sustainability and effectiveness of the program. Consequently, the erosion of relationships and trust may hinder the effectiveness of CHWs in their role as a foundational component of the Philippines’ plan for the implementation of UHC.

### Non-state actors can create short-term supports for CHW programs

Across the two regions in our study, non-state actors contributed to the diversity of implementation and effectiveness of CHW programs. In some communities, CHW training was directly provided by NGOs, while in others, NGOs financially sponsored training; however, not all CHWs had access to training provided by NGOs. As mentioned, partnerships between CHW programs and NGOs were seen by one administrator as a “golden opportunity” to improve community-level health outcomes. Thus, in communities without access to NGO support, the training options of CHWs were limited to training provided by the local or regional governments. Existing research has found that collaboration between multiple partners, including state and non-state actors, can contribute to the successful implementation of CHW programs [15]. Additionally, co-creating programs and sharing responsibility between partners (e.g., NGOs, local governments, and civil society) has been identified as an important way of enhancing community ownership of CHW interventions [15]. However, there are also concerns that dependence on NGOs can minimise state responsibility and lead to fragmented funding, training, supervision, and monitoring within CHW programs [9, 16, 21, 49].

In a devolved health system, the partnership between local governments and non-state actors can help CHW programs develop the capacity to efficiently identify and creatively respond to local community needs; establish successful approaches to recruiting, training, and retaining CHWs; and create monitoring programs informed by both communities and public health systems [15, 16]. Involving NGOs in CHW programs can also help address resource constraints faced by local governments [16]. As efforts turn toward the implementation of UHC in the Philippines and elsewhere, we see partnerships between state and non-state actors as a potential short-term measure to support CHW programs and bolster local government capacity during this time of transition to UHC.

### Opportunities to enhance CHW program effectiveness within a decentralized health system

Although we found that decentralization contributed to a number of governance challenges in the Philippines’ CHW programs, it is important to reflect on the potential benefits of decentralization for achieving UHC, in addition to the role of CHWs within this process. Many policy makers believe that, if implemented correctly, decentralization has the ability to positively contribute to enhancing local “authority, autonomy, accountability, and community participation” in health care ([50], p.1). Health care decentralization can foster communication and accountability through the involvement of multiple centres of governance (i.e., communities and regional or national health authorities) [51]. Accountability positively influences health care delivery, the fulfilment of CHWs’ roles, and the treatment of patients [52]. Decentralization can also empower local actors to make decisions about health care, which can help address inequalities in the distribution of resources and enhance local input, participation, and control [53–55]. By enhancing mechanisms for local decision-making, decentralization can reduce disparities between groups or regions with competing priorities, such as rural-urban divides [55]. Additionally, in many LMICs, decentralization has contributed to more widely available and accessible services, generated more resources for health, and improved health outcomes [54].

Thus, as CHWs have been identified as key to enhancing the equity and effectiveness of health care delivery [10, 15, 16, 18], improving local control of CHW programs has the potential to positively contribute to the implementation of UHC. In order to realize the full role CHW programs can play in achieving UHC – such as the ability of programs to extend the reach of formal health care providers, enhance access and equity of health services, and improve individual and community-level health outcomes – more research is needed to explore the governance challenges and opportunities of CHW programs in various decentralized health systems. We suggest that drawing on and taking seriously the experiences of CHWs (e.g., including their perspectives in program evaluations and working closely with regional CHW associations to guide policies impacting CHWs) as well as individuals involved in the implementation of CHW programs, can positively contribute to informing how CHWs and CHW programs can be effectively leveraged in efforts to achieve UHC.

Furthermore, we suggest that within a decentralized health care system, CHW accreditation can act as a key mechanism to enhance the positive outcomes of CHW programs, including the potential benefits to CHWs. The Government of the Philippines mandated CHW accreditation within the BHW Act (1995) [35]; however, our findings indicate that accreditation of CHWs was not consistently operationalized at a local level (i.e., CHWs employed at the *barangay* level were often not accredited). Although there is limited research on the benefits of accreditation for CHWs in the Philippines, research on CHW programs in other LMICs (e.g., Ethiopia, Iran, Kenya, and Afghanistan) has identified accreditation as a potential tool to improve the morale, job security, career opportunities, legitimacy, and social status of CHWs [56, 57]. For example, in our study we found that CHW accreditation can act as a buffer against the influence of local politics, improving the retention and stability of CHW programs. Additionally, accreditation can enhance oversight and standardization of the performance of CHWs [57], and lead to more reliable implementation of CHW programs across settings, which can, in turn, help improve accountability at various levels within a decentralized health care system [52].

Overall, accreditation must balance national priorities, such as UHC, with the capacity of local and regional health authorities to operationalize national CHW guidelines. To realize the potential of CHW accreditation in the Philippines, there is an opportunity to reinforce existing legislation (i.e., the BHW Act) and build capacity across local governments to enhance understanding of and commitment to adequately resourcing and training CHWs. More specifically, accreditation can be used as a tool to encourage efforts by local governments to improve health services and outcomes among citizens through enhancing the consistency and quality of CHW programs. However, accreditation processes must also be sensitive and responsive to gender and other socio-cultural factors that shape the recruitment and retention of CHWs. Indeed, accreditation should bring further legitimacy and stability to the CHW role, in addition to greater recognition of the value and contributions of CHWs for their communities.

### Limitations

While this study includes participants from six cities across two provinces in the Philippines, the experiences and insights shared are not necessarily reflective of all CHWs or CHW programs across the Philippines. This limitation is particularly important in the context of a decentralized health system where other LGUs may provide different resources or supports for CHWs. However, our study provides an important foundation for future research on the connections between health system decentralization and the governance of CHW programs in the context of the implementation of UHC.

## Conclusion

Globally, there has been a push toward achieving UHC in many LMICs, with CHWs recognized as an important resource for the implementation of UHC. Drawing on the experiences of CHWs and CHW administrators from two provinces in the Philippines, this study demonstrated how health system decentralization can create governance challenges for CHW programs in the midst of efforts to achieve UHC. We showed how variations across and within settings in terms of the administration and functioning of CHW programs, in addition to the concentration and use of power at the local level, can either facilitate (e.g., sufficient resources allocated for CHWs in *barangays*) or hinder (e.g., removal of non-accredited CHWs with the change of local administration) the ability of CHWs to effectively engage in their work.

Health system decentralization can contribute to the uneven implementation of national health priorities at the local level, which can have consequences for the functioning of CHW programs and the experiences of CHWs. At the same time, health system decentralization can also contribute to enhanced autonomy, accountability, and local participation in health care. To leverage the benefits of health system decentralization to enhance the functioning of CHW programs, there is a need to balance national priorities with the capacity of local and regional health authorities to operationalize national CHW guidelines. In the context of the implementation of UHC in the Philippines and in other LMICs, CHW accreditation represents a promising strategy for promoting national health priorities through improving the consistency and quality of CHW programs across settings. By strengthening accreditation processes, in addition to broader efforts to enhance the capacity and commitment of local governments to adequately resource CHW programs, CHWs will be well-positioned to meaningfully contribute to UHC.

## Supplementary Information

**Additional file 1.**

## Data Availability

The data that support the findings of this study are available on reasonable request from the corresponding author. The data are not publicly available to maintain the confidentiality of research participants.

## Abbreviations

BHW: Barangay Health Worker
BNS: Barangay Nutrition Scholar
CHW: Community Health Worker
DOH: Department of Health
LGU: Local Government Unit
LMIC: Low- and Middle-Income Countries
NGO: Non-Governmental Organization
UHC: Universal Health Coverage

## Footnotes

1 We define CHW program governance as the implementation, administration, and functioning of a CHW program within a country.

2 A *barangay* refers to the smallest political unit in the Philippines [25].

3 A *barangay* captain is the highest elected official in a *barangay.*

4 *Barangays* are informally divided into smaller zones known as *puroks* at the sub-village level [25].

## References

[1] United Nations (2020). The sustainable development goals.

[2] Tulenko K, Mgedal S, Afzal MM, Frymus D, Oshin A, Pate M, Quain E, Pinel A, Wynd S, Zodpey S (2013). Community health workers for universal health-care coverage: from fragmentation to synergy. Bull World Health Organ.

[3] Gilmartin C (2017). Community health workers: a Prioritiy for universal health coverage?. Management Sciences for Health.

[4] Perry H, Crigler L, Lewin S, Glenton C, LeBan K, Hodgins S (2017). A new resource for developing and strengthening large-scale community health worker programs. Hum Resour Health.

[5] Scott K, Beckham S, Gross M, Pariyo G, Rao KD, Cometto G, Perry HB (2018). What do we know about community-based health worker programs? A systematic review of existing reviews on community health workers. Hum Resour Health.

[6] Kok MC, Broerse JE, Theobald S, Ormel H, Dieleman M, Taegtmeyer M (2017). Performance of community health workers: situating their intermediary position within complex adaptive health systems. Hum Resour Health.

[7] Zulu JM, Kinsman J, Michelo C, Hurtig A-K (2014). Integrating national community-based health worker programmes into health systems: a systematic review identifying lessons learned from low-and middle-income countries. BMC Public Health.

[8] Ruano AL, Hernández A, Dahlblom K, Hurtig AK, San Sebastián M (2012). ‘It’s the sense of responsibility that keeps you going’: stories and experiences of participation from rural community health workers in Guatemala. Arch Public Health.

[9] Kok MC, Kane SS, Tulloch O, Ormel H, Theobald S, Dieleman M, Taegtmeyer M, Broerse JE, de Koning KA (2015). How does context influence performance of community health workers in low-and middle-income countries? Evidence from the literature. Health Res Policy Syst.

[10] McCollum R, Gomez W, Theobald S, Taegtmeyer M (2016). How equitable are community health worker programmes and which programme features influence equity of community health worker services? A systematic review. BMC Public Health.

[11] Rafiq MY, Wheatley H, Mushi HP, Baynes C (2019). Who are CHWs? An ethnographic study of the multiple identities of community health workers in three rural districts in Tanzania. BMC Health Serv Res.

[12] Schneider H (2019). The governance of national community health worker programmes in low-and middle-income countries: an empirically based framework of governance principles, purposes and tasks. Int J Health Policy Manag.

[13] Lehmann U, Gilson L (2013). Actor interfaces and practices of power in a community health worker programme: a south African study of unintended policy outcomes. Health Policy Plan.

[14] Condo J, Mugeni C, Naughton B, Hall K, Tuazon MA, Omwega A, Nwaigwe F, Drobac P, Hyder Z, Ngabo F (2014). Rwanda’s evolving community health worker system: a qualitative assessment of client and provider perspectives. Hum Resour Health.

[15] Naimoli JF, Perry HB, Townsend JW, Frymus DE, McCaffery JA (2015). Strategic partnering to improve community health worker programming and performance: features of a community-health system integrated approach. Hum Resour Health.

[16] Asweto CO, Alzain MA, Andrea S, Alexander R, Wang W (2016). Integration of community health workers into health systems in developing countries: opportunities and challenges. Family Med Comm Health.

[17] Lewin S, Lehmann U, Crigler L, Glenton C, Hodgins S, LeBan K, Lewin S, Perry H (2014). Governing large-scale community health worker programs. Developing and strengthing community health worker programs at scale: a reference guide and case studies for program managers and policy makers.

[18] Schneider H, Nxumalo N (2017). Leadership and governance of community health worker programmes at scale: a cross case analysis of provincial implementation in South Africa. Int J Equity Health.

[19] Glenton C, Scheel IB, Pradhan S, Lewin S, Hodgins S, Shrestha V (2010). The female community health volunteer programme in Nepal: decision makers’ perceptions of volunteerism, payment and other incentives. Soc Sci Med.

[20] Maes K, Kalofonos I (2013). Becoming and remaining community health workers: perspectives from Ethiopia and Mozambique. Soc Sci Med.

[21] Swartz A (2015). Colvin CJ: ‘It’s in our veins’: caring natures and material motivations of community health workers in contexts of economic marginalisation. Crit Public Health.

[22] Prieto-Carolino A, Mamauag BL (2019). Pagdipara: caring work by poor elderly women in coastal communities in Iloilo, Philippines. Asian J Women’s Stud.

[23] UHC Act in the Philippines: A new dawn for health care [https://www.who.int/philippines/news/feature-stories/detail/uhc-act-in-the-philippines-a-new-dawn-for-health-care]. Accessed 20 Nov 2020.

[24] Community health workers and volunteers: The most effective path to UHC [https://www.globalhealthnow.org/2019-04/community-health-workers-and-volunteers-most-effective-path-uhc]. Accessed 20 Nov 2020.

[25] Matthies A (2017). Community-based disaster risk Management in the Philippines: achievements and challenges of the Purok system. Austrian J South-East Asian Stud.

[26] Sison O, Castillo-Carandang N, Ladia MA, Sy R, Punzalan FE, Llanes EJ, Reganit PF, Velandria F, Gumatay WA (2019). Prevalence of metabolic syndrome and cardiovascular risk factors among community health Workers in Selected Villages in the Philippines. J ASEAN Federation Endocrine Soc.

[27] Barangay Nutrition Scholar (BNS) Program [https://www.doh.gov.ph/health-program/BNS]. Accessed 20 Nov 2020.

[28] Abrigo MRM, Ortiz DAP (2018). Devolution of health services, fiscal decentralization, and antenatal care in the Philippines. PIDS Discussion Paper Series. vol. Report No. DP 2018–42: Philippine Institute for Development Studies.

[29] Cuenca JS (2018). Health Devolution in the Philippines: Lessons and Insights. PIDS Discussion Paper Series. vol. No. DP 2018–36: Philippine Institute for Development Studies.

[30] Cuevas PRF, Calalang CF, Reyes DJAD, Rosete MAL (2017). The impact of decentralization of the Philippines’ public health system on health outcomes. Int J Adv Res Technol.

[31] Langran IV (2011). Decentralization, democratization, and health: the Philippine experiment. J Asian Afr Stud.

[32] Espino F, Beltran M, Carisma B (2004). Malaria control through municipalities in the Philippines: struggling with the mandate of decentralized health programme management. Int J Health Plann Manag.

[33] Dayrit MM, Lagrada LP, Picazo OF, Pons MC, Villaverde MC. The Philippines Health System Review. Vol. 8 No. 2. New Delhi: World Health Organization, Regional Office for SouthEast Asia; 2018.

[34] Leopando ZE. Universal Health Care: The Philippine Experience. 2011. https://www.slideshare.net/Healthandlabour/v2-philippines-uhc-doh-the-hague. Accessed 20 Nov 2020.

[35] Congress of the Philippines: Republic Act no. 7883: An act granting benefits and incentives to accredit barangay health workers and for other purposes. In. Metro Manila, Republic of the Philippines; 1995. Retrived from: http://legacy.senate.gov.ph/lisdata/3041327193!.pdf

[36] Yamashita T, Suplido SA, Llave C, Tuliao MTR, Tanaka Y, Matsuo H (2015). Understanding postpartum healthcare services and exploring the challenges and motivations of maternal health service providers in the Philippines: a qualitative study. Trop Med Health.

[37] Sumaylo DJF (2013). Information delivery in the provision of barangay health Services in Barangay Dawis, Digos City, Philippines. J Asia Pacific Stud.

[38] Matsumoto-Takahashi ELA, Kano S (2016). Evaluating active roles of community health workers in accelerating universal access to health services for malaria in Palawan, the Philippines. Trop Med Health.

[39] Sy TRL, Padmawati RS, Baja ES, Ahmad RA (2019). Acceptability and feasibility of delegating HIV counseling and testing for TB patients to community health workers in the Philippines: a mixed methods study. BMC Public Health.

[40] Endrina-Ignacio MS (2015). Assessment of the preparedness of the barangay nutrition scholars (BNS) in implementing barangay nutrition action plan in selected municipalities in Ifugao, Bulacan, and Siquijor. Philippine J Health Res Dev.

[41] Ramiro LS, Castillo FA, Tan-Torres T, Torres CE, Tayag JG, Talampas RG, Hawken L (2001). Community participation in local health boards in a decentralized setting: cases from the Philippines. Health Policy Plan.

[42] Closser S, Maes K, Gong E, Sharma N, Tesfaye Y, Abesha R, Hyman M, Meyer N, Carpenter J (2020). Political connections and psychosocial wellbeing among Women's development Army leaders in rural Amhara, Ethiopia: Towards a holistic understanding of community health workers’ socioeconomic status. Social Sci Med.

[43] Mallari E, Lasco G, Sayman DJ, Amit AML, Balabanova D, McKee M, Mendoza J, Palileo-Villanueva L, Renedo A, Seguin M (2020). Connecting communities to primary care: a qualitative study on the roles, motivations and lived experiences of community health workers in the Philippines. BMC Health Serv Res.

[44] Tuyisenge G, Crooks VA, Berry NS (2020). Using an ethics of care lens to understand the place of community health workers in Rwanda's maternal healthcare system. Soc Sci Med.

[45] Steege R, Taegtmeyer M, McCollum R, Hawkins K, Ormel H, Kok M, Rashid S, Otiso L, Sidat M, Chikaphupha K (2018). How do gender relations affect the working lives of close to community health service providers? Empirical research, a review and conceptual framework. Soc Sci Med.

[46] Mumtaz Z, Salway S, Nykiforuk C, Bhatti A, Ataullahjan A, Ayyalasomayajula B (2013). The role of social geography on lady health Workers’ mobility and effectiveness in Pakistan. Soc Sci Med.

[47] Twumasi PA, Freund PJ (1985). Local politicization of primary health care as an instrument for development: a case study of community health workers in Zambia. Soc Sci Med.

[48] Nxumalo N, Goudge J, Thomas L (2013). Outreach services to improve access to health care in South Africa: lessons from three community health worker programmes. Glob Health Action.

[49] Ndima SD, Sidat M, Ormel H, Kok MC, Taegtmeyer M (2015). Supervision of community health workers in Mozambique: a qualitative study of factors influencing motivation and programme implementation. Hum Resour Health.

[50] Tolera H, Gebre-Egziabher T, Kloos H (2019). Public health service delivery in a decentralized system: a qualitative study of the perception of health providers and community members in Gida Ayana Woreda, Western Ethiopia. Global J Med Res.

[51] Abimbola S, Baatiema L, Bigdeli M (2019). The impacts of decentralization on health system equity, efficiency and resilience: a realist synthesis of the evidence. Health Policy Plan.

[52] Schaaf M, Fox J, Topp SM, Warthin C, Freedman LP, Robinson RS, Thiagarajan S, Scott K, Maboe T, Zanchetta M (2018). Community health workers and accountability: reflections from an international “think-in”. Int J Equity Health.

[53] Zarychta A (2020). Making social services work better for the poor: evidence from a natural experiment with health sector decentralization in Honduras. World Dev.

[54] Muñoz DC, Amador PM, Llamas LM, Hernandez DM, Sancho JMS (2017). Decentralization of health systems in low and middle income countries: a systematic review. Int J Public Health.

[55] Brennan E, Abimbola S (2020). Understanding and progressing health system decentralisation in Myanmar. Global Security: Health Sci Policy.

[56] Najafizada SAM, Labonté R, Bourgeault IL (2019). HRH dimensions of community health workers: a case study of rural Afghanistan. Hum Resour Health.

[57] Onono M, Abdi M, Opondo I, Okung'u J, Asadhi E, Nyamai R, Karimurio L, Okoth P, Qazi SA (2018). Using the RE-AIM framework to evaluate the implementation of integrated community case management in Kenya. Acta Paediatr.

