# Supplementary material for: Governance of community health worker programs in a decentralized health system: a qualitative study in the Philippines
Source: BMC Health Serv Res. 2021 May 12;21:451. doi: 10.1186/s12913-021-06452-x (PMC8114679; doi:10.1186/s12913-021-06452-x)
Supplement: Supplementary file 1 — Additional file 1. [file 12913_2021_6452_MOESM1_ESM.docx]

**Governance of community health worker programs in a decentralized health system: a qualitative study in the Philippines**

Warren Dodd^1*^, Amy Kipp^1^, Bethany Nicholson^1^, Lincoln Leehang Lau^2^, Matthew Little^3^, John Walley^4^, Xiaolin Wei^5^

1 School of Public Health and Health Systems, University of Waterloo, Waterloo, Canada

2 International Care Ministries, Manila, Philippines

3 School of Public Health and Social Policy, University of Victoria, Victoria, Canada

4 Nuffield Centre for International Health and Development, University of Leeds, Leeds, United Kingdom

5 Dalla Lana School of Public Health, University of Toronto, Toronto, Canada

**Warren Dodd**

*Corresponding Author

School of Public Health and Health Systems, University of Waterloo,

200 University Ave. W., Waterloo, Ontario, Canada, N2L 3G1

wdodd@uwaterloo.ca

**Table S1.** Semi-structured interview guide exploring governance of community health workers programs in Negros Occidental and Negros Oriental, Philippines

| *Personal* | Position: | Years in practice: | Age: | Education: | City vs. Brgy.  Volunteer period? |
| --- | --- | --- | --- | --- | --- |
| *Background* | How did you become a BHW/BNS?  Why do you think you were selected/hired? What were the requirements?  How long do you anticipate working as a BHW/BNS? | | | | |
|  | Can you describe a typical day/week?  How often do you see the RHU Nurse/Midwife throughout your day? | | | | |
|  | What are your main duties responsibilities in the health station? In the community? | | | | |
|  | What training did you receive to become a BHW/BNS?  Is there a cost? How much? Who pays? | | | | |
|  | When did you receive your initial BHW/BNS training? | | | | |
|  | Where did you receive your training? Why there?  Who delivered your training?  What was the format? | | | | |
|  | Did you receive any illness or treatment-specific training?  -tuberculosis  -immunizations  -nutrition  -pediatric care  -prenatal care & maternal/child health  -chronic disease screening & management | | | | |
|  | Who decides which BHWs receive training? Is the barangay captain involved in the decision?  Is training mandatory prior to employment as a BHW or BNS?  Are there times when BHWs do not report for work or work at the barangay captain’s office because they have not received the appropriate training?  (probe around political nature/dimensions of training) | | | | |
| *Continuing education and support* | Do you think the initial training you received has prepared you for your role? How much have you had to learn on the job? | | | | |
|  | Have you received any additional training since you received your initial training?  How are you updated about best practices and advancements or changes in protocol?  How often is additional training available or offered? | | | | |
|  | Do you have a manual or guidelines to reference from your training? How often are these manuals updated? | | | | |
|  | Is there additional training for specific health topics? What is the format? (preceptorship, lecture, observation, etc.) | | | | |
|  | IF YES: If there are opportunities for additional training and/or continuing education. Who pays for this?  What types of costs are incurred (e.g., transportation, lost days of work, staying overnight somewhere?)  Is this training required or voluntary (i.e. personal interest)? | | | | |
|  | Does compensation differ for BHW/BNS? Is it significant?  What are the factors that influence compensation for BHWs and BNS?  Experience?  Education level?  City vs. Barangay? | | | | |
|  | Are there any other types of support that you receive for your work (transportation stipend, etc.) | | | | |
| *Barangay Nutrition Scholars (BNS)* | When did the BNS program start? | | | | |
|  | Why did the BNS program start? | | | | |
|  | Is there any specialized training that BNS receive that BHWs do not receive (or vice versa) | | | | |

Brgy = Barangay; BHW = barangay health worker; BNS = barangay nutrition scholar
